# Interface Intermixing in Type II InAs/GaInAsSb Quantum Wells Designed for Active Regions of Mid-Infrared-Emitting Interband Cascade Lasers

**DOI:** 10.1186/s11671-015-1183-x

**Published:** 2015-12-07

**Authors:** Marcin Motyka, Grzegorz Sęk, Krzysztof Ryczko, Mateusz Dyksik, Robert Weih, Gilles Patriarche, Jan Misiewicz, Martin Kamp, Sven Höfling

**Affiliations:** Laboratory for Optical Spectroscopy of Nanostructures, Department of Experimental Physics, Faculty of Fundamental Problems of Technology, Wrocław University of Technology, Wybrzeże Wyspiańskiego 27, Wrocław, Poland; Technische Physik and Wilhelm-Conrad-Röntgen-Research Center for Complex Material Systems, University of Würzburg, Am Hubland, 97074 Würzburg, Germany; Laboratoire de Photonique et de Nanostructures, CNRS, Université Paris-Saclay, Route de Nozay, 91460 Marcoussis, France; School of Physics and Astronomy, University of St. Andrews, North Haugh, KY16 9SS St. Andrews, UK

**Keywords:** Type II GaIn(As)Sb/GaSb, QW interface profile, Intermixing, Interband cascade lasers, FTIR spectroscopy, EDX spectra

## Abstract

The effect of interface intermixing in W-design GaSb/AlSb/InAs/Ga_0.665_In_0.335_As_x_Sb_1 − x_/InAs/AlSb/GaSb quantum wells (QWs) has been investigated by means of optical spectroscopy supported by structural data and by band structure calculations. The fundamental optical transition has been detected at room temperature through photoluminescence and photoreflectance measurements and appeared to be blueshifted with increasing As content of the GaInAsSb layer, in contrast to the energy-gap-driven shifts calculated for an ideally rectangular QW profile. The arsenic incorporation into the hole-confining layer affects the material and optical structure also altering the InAs/GaInAsSb interfaces and their degree of intermixing. Based on the analysis of cross-sectional transmission electron microscopy images and energy-dispersive X-ray spectroscopy, we could deduce the composition distribution across the QW layers and hence simulate more realistic confinement potential profiles. For such smoothed interfaces that indicate As-enhanced intermixing, the energy level calculations have been able to reproduce the experimentally obtained trend.

## Background

The mid-infrared spectral range is of crucial importance in gas-sensing applications due to the existence of strong absorption lines within this region. Hence, a laser emitting above 3 μm is the main component of modern highly efficient optical sensor systems for detection of harmful gases, such as the following: hydrocarbons, CO_2_, and NH_3_ [1]. For these applications, the radiation source is usually required to operate in a continuous wave, as well as single mode at room temperature, and at a very certain wavelength—one that matches the respective vibrational-rotational transitions in the target molecules. Several possible solutions exist for how to construct semiconductor lasers in this spectral range. The first, based on type I quantum well structures, is limited to below 4 μm due to the accessible band gaps and band offsets in a combination of quaternary GaInAsSb and quinary AlGaInAsSb alloys of GaSb-based devices [2, 3]. The second type are quantum cascade lasers (QCLs) which require substantial band discontinuity in the conduction band of the active quantum wells [4, 5] making the fabrication challenging, with the additional drawback of large threshold currents and high power consumption. In that respect, the idea to employ the so-called interband cascade lasers (ICLs) [6] utilizing type II InAs/GaInSb quantum wells in the active region [7, 8] is very promising. One of the advantages of this solution is the ease of spectral tunability achieved via variation of the thickness of the conduction-band-well layer [9]. Furthermore, the ICLs can offer lower threshold currents and much lower electrical power consumption than the QCLs [10].

Optimization of type II quantum well (QW) structures, serving as the active region, is crucial for final device performance. One of the concepts of how to improve the performance and expand the possible ICL emission range is to replace the ternary GaInSb valence band well material in the InAs/GaInSb/InAs sequence by a quaternary layer of GaInAsSb [11]. Intrinsic limitations of the ICLs with GaInSb hole-confining layers are related to carrier losses [12] or the strain of GaInSb with respect to the substrate [13]. Replacing the ternary GaInSb material of the valence band well with the quaternary GaInAsSb layer may help to reduce this strain, as well as allow increasing of the indium content in this layer. It has also been demonstrated that such quaternary alloys replacing GaInSb can help to enhance the optical transition oscillator strength [14]. However, some shortcomings of this solution have been recognized as the incorporation of arsenic atoms into the GaInSb layer may cause some structural changes [15].

In this work, we investigate the interface intermixing in the type II structures of InAs/GaIn(As)Sb/InAs and its consequences to the QW electronic structure and optical properties. We compare the results of photoluminescence (PL) and photoreflectance (PR) measurements with numerical calculations. To calculate the electronic structure in type II W-design QWs, we use the eight-band k·p Hamiltonian based on Luttinger and Kohn theory, defined for the [001] growth direction [16, 17]. Our eight-band model includes the strain effects. The carrier wave functions and the subband energies have been determined by numerically solving the Schrödinger equation and employing the finite difference method. We obtained the potential profile of the quantum wells by using well-known approximations for the error function profile, characteristic for the interdiffused interfaces (to be discussed later). Cross-sectional scanning transmission electron microscopy (STEM) images, energy-dispersive X-ray spectroscopy (EDX), and high-resolution X-ray diffraction (HR-XRD) data demonstrate that incorporation of arsenic into the holes’ confinement layer of the “W”-shaped structure causes significant intermixing at the InAs/GaInAsSb interface.

## Methods

The investigated structures were grown on (100)-oriented GaSb substrates by a solid source molecular beam epitaxy system equipped with valved cracker cells for both antimony and arsenic. The type II QWs have been designed in the shape of the “W-like” QWs and as such demonstrate larger electron-hole wave function overlap compared to a common, single InAs layer type II configuration and hence are more prospective from the application point of view [9, 11, 14]. The studied QWs consist of two InAs layers (1.4 nm thick) confining electrons and one Ga_0.665_In_0.335_As_x_Sb_1 − x_ layer (3.5 nm) in between for the confinement of holes. This core part is surrounded by 2.5-nm-thick AlSb barriers. In order to enhance the overall optical response, the wells have been repeated five times and separated from each other by 20 nm of GaSb. The growth is initiated with a thermal oxide desorption at a substrate temperature of 580 °C under Sb flux. Subsequently, the temperature is ramped down to 485 °C, and the growth of the 300-nm-thick buffer layer is initiated at a growth rate of 1000 nm/h and a V–III beam equivalent pressure (BEP) ratio of 7. The substrate temperature is ramped down by the end of the buffer layer and stabilized in order to ensure a stable substrate temperature of 450 °C during the W-QW growth. The InAs and the ternary layers were grown at a rate of 280 nm/h and a V–III BEP ratio of 10 and a growth rate of 830 nm/h and a V–III BEP ratio of 7, respectively. During the growth of the W-QWs, no specific interface type was forced by shutter sequencing. In the case of the quaternary layers, the As valve was ramped 0.8 s before the end of the first InAs layer and 0.8 s before the end of the quaternary layer to ensure sufficient flux to achieve the desired stoichiometry. The entire structure is followed by a 25-nm GaSb cap. Two structures differing in the composition of the hole-confining layers have been investigated: sample A with a ternary Ga_0.665_In_0.335_Sb layer, i.e., without arsenic, and sample B with Ga_0.665_In_0.335_As_0.1_Sb_0.9_. The quaternary alloy was calibrated in a series of test structures which were grown and characterized via HR-XRD prior to the W-QW samples. The Sb flux was kept constant for both samples which, by adding As, leads to a slightly higher V–III ratio within the quaternary hole quantum well of sample B. Preliminary optical and structural properties of these samples have already been investigated previously [15].

**Fig. 1:**
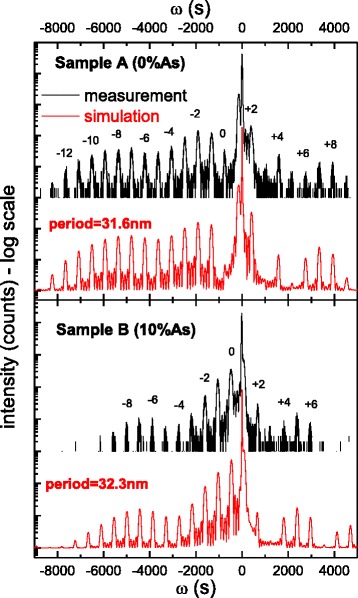
HR-XRD spectra for the type II QWs with various As contents: ternary layer of GaIn(As)Sb with no arsenic (*Sample A*) and 10 % of As (*Sample B*). The shift of the envelope towards a larger angle indicating a strain reduction in the W-QW is clearly visible

HR-XRD measurements have been performed to confirm the incorporation of arsenic atoms into the GaInSb layers. This result is presented in Fig. 1, which shows scans made for the two investigated samples. The GaSb substrate peak (the highest) is clearly visible on both scans together with a number of satellite peaks connected with the periodic structure of the layers. For sample A, satellites up to the 13th order can be observed which indicates a high crystal quality and sharpness of the interfaces. The number of observable peaks is reduced for sample B which suggests partial crystal degradation due to, for instance, smearing of the interfaces. The envelope of the periodic peaks also shifts towards a larger angle for sample B (comparing to sample A), which means that layers are less strained with respect to the substrate as a result of arsenic incorporation (reducing the lattice mismatch with respect to GaSb substrate). The respective fitting of the X-ray curves by the simulations (shown in red color) allowed for the determination of layer thicknesses which agreed very well with the nominal ones. The comparison between simulated and measured curves gives the fundamental structure period (a sum of QW layer thicknesses and the separating barrier) of 31.6 nm for sample A and 32.3 nm for sample B confirming that the real QW parameters are indeed close to the nominal ones. The small discrepancies are rather within the accuracy of the fit.

To probe the structural quality of the layers, cross-sectional STEM measurements have been performed in the high-angle annular dark field (HAADF) mode. Specimens from the samples were prepared for STEM using conventional mechanical polishing (down to a thickness of approximately 20 μm) and argon ion milling utilizing the precision ion polishing system of Gatan and following the <110> crystallographic direction. This is obtained thanks to the size of the ion beam (approximately 1 mm large) and utilization of the natural nonuniformity of the etching. The process is stopped when a hole appears at the center of the thin foil. Sample thickness is increasing gradually, as one moves away from the center. Parts of the sample that are thin enough to be transparent to electrons are located around the hole. Before the ion-thinning process, the thin sample was glued onto a copper ring. The final specimen thickness was between 25 and 50 nm. Prior to STEM imaging, the specimens were cleaned using an argon plasma cleaner. HAADF STEM and energy-dispersive X-ray spectroscopy (EDX) were performed in an aberration-corrected JEOL 2200FS microscope, operating at 200 kV with a probe current of 150 pA and the full width at the half maximum (FWHM) of the probe being 0.12 nm. The convergence half-angle of the probe was 30 mrad, and the detection inner and outer half-angles for the STEM images were 100 and 170 mrad, respectively. In order to probe the composition across the layers, the EDX technique has been used, based on the intensity ratio of the respective X-ray spectral lines. The acquisition time for each EDX spectrum was 60 s, during which no drift in the position of the electron beam was observed. Further experimental details can be found elsewhere [18, 19].

In order to measure photoluminescence (PL) and photoreflectance (PR) spectra [20–22] in that spectral range, we utilized a setup based on a Fourier transform and vacuum spectrometer Bruker Vertex 80v operated in step-scan mode and equipped with an external chamber for experiments employing an additional mechanically modulated laser beam. In this case, a liquid-nitrogen-cooled InSb photodiode was used as a detector. In both measurement configurations, the pump beam was provided by the 660-nm line of a semiconductor laser diode, modulated with a frequency of 275 Hz. Phase-sensitive detection of the optical response was performed using a lock-in amplifier.

## Results and Discussion

**Fig. 2:**
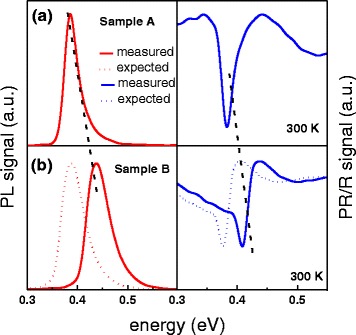
Room temperature photoluminescence (*red lines*—*left part*) and photoreflectance (*blue lines*—*right part*) spectra of ground state type II “W”-shaped quantum well transitions measured for sample A (**a**) and sample B (**b**). *Dotted lines* show the expected spectra at the calculated nominal transition energy

**Fig. 3:**
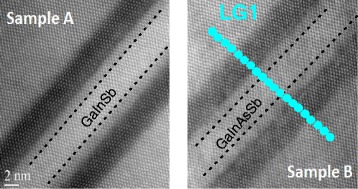
HAADF-STEM images for samples A and B. *Blue circles* represent points taken to EDX analysis. All the samples were prepared following a <110> zone axis (note: the zone axis is the direction normal to the image). EDX profiles are along the <001> direction (the growth axis). This means that the <−110> direction is along the interfaces

It has been revealed previously [14, 15] that incorporation of arsenic into the GaInSb layer of the W-design II QW system provides additional possibilities in terms of band alignment and strain tailoring and allows for the enhancement of oscillator strength of the active type II transition. All those can generally be considered as beneficial from a band structure engineering point of view and also help to improve the performance of interband cascade lasers. On the other hand, however, the addition of arsenic seems to have generated interface localized states, which can trap the carriers decreasing the radiative efficiency of emission from this kind of quantum wells [15]. Hereby, we report on the observation of another side effect, related to the compositional changes in the hole-confining layer, affecting the entire type II system, which can be crucial for properly predicting the electronic structure, as well as for device fabrication with the demanded emission wavelength. In Fig. 2, room temperature (300 K)-normalized PL and PR spectra measured for the investigated samples are presented. The single peaks in the PL spectra are related to the ground state type II transition in the QWs. The same transition is detected in the PR spectra. These lines are related to the 1e–1h transition, where 1e means the first electron level confined in the InAs layers and 1h the first hole level confined in the GaIn(As)Sb layer. The energy of the transition for sample B is shifted towards blue (shown by black dashed lines) after incorporation of As into the valence band well layer. The dotted lines in Fig. 2 represent the theoretically predicted position of these spectra, i.e., for an ideally rectangular QW profile [23–26], which results from several composition-related effects. The primary influence on the ground state transition energy shift is attributed to the As-induced band gap change [27]. Important contributions come from strain modification in the GaIn(As)Sb layer and changes of the confinement potential; that is, in the type II system with two InAs layers, the change of the GaInAsSb energy gap entails also a change of the barrier height and the resulting coupling. A reasonable agreement between the theory and experiment has only been demonstrated for sample A, with the ternary GaInSb layer. However, the energy shift for sample B in the experiment is opposite to that predicted by the model. The experimental behavior may suggest that incorporation of arsenic into the GaInSb layer could change the profile of the QW in such a way as to result in electronic structure modification, eventually causing a blueshift instead of a redshift. The measured energy blueshift cannot be explained by QW layer width changes because, as it was previously shown above, the obtained periods provided by XRD spectra are almost the same for both samples and very close to the nominal values. In our previous article [15], we have already demonstrated that arsenic can have a strong impact on the InAs/GaInAsSb interfaces. Therefore here, for better understanding of these effects, we have analyzed the atomic resolution HAADF-STEM images of the investigated samples in the cross-sectional plane perpendicular to the interfaces. This image is presented in Fig. 3. The interfaces are very sharp for the ternary layer structure (sample A) and already a bit smeared out for the As-containing structure (sample B) (the mentioned interfaces are shown by dashed black lines). Although the interfaces between the InAs and GaIn(As)Sb layers can barely be seen for both the cases, due to the natural low image contrast for these materials, these STEM images already suggest an enhanced intermixing after the addition of arsenic.

**Fig. 4:**
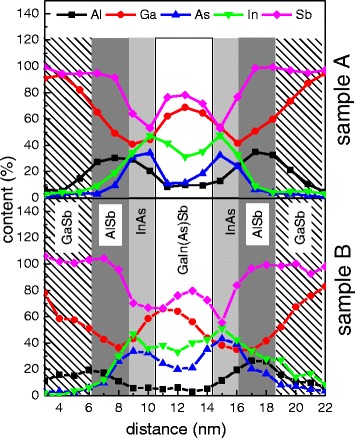
EDX profiles of the investigated samples (*solid symbols*). *Various colors* represent different elements in subsequent layers. In the background, *colored areas* demonstrate the nominal thickness of each layer

In order to interpret the results more quantitatively, we have also performed EDX analysis of the samples. The blue trace over the sample’s STEM cross-sectional plane image (example shown for sample B—right side of Fig. 3) corresponds to points used for EDX analysis. A single spot in this analysis is about 1.5 nm wide. The results are presented as full symbols in Fig. 4. Despite the convolution with the probe size, and hence quite a limited resolution of the composition determination when compared to the thicknesses of the layers, we can observe a clear variation of the composition inside the structure. Various elements are represented by different colors in Fig. 4, whereas the colors of the background indicate the nominal layer thicknesses. Although the resolution of the composition contrast is low, comparing the results for samples A and B allows us to see that the smoothening of the interfaces for the latter, i.e., enhancement of the intermixing of the atoms and finally creation of the less clearly defined interfaces between layers, can be observed. The atoms’ intermixing is the reason for the electronic structure modification, leading to the confined level shifts and, consequently, a blueshift of the ground state transition energy for the sample with a thin GaInAsSb layer (used for hole confinement) with respect to the reference structure containing a GaInSb layer.

**Fig. 5:**
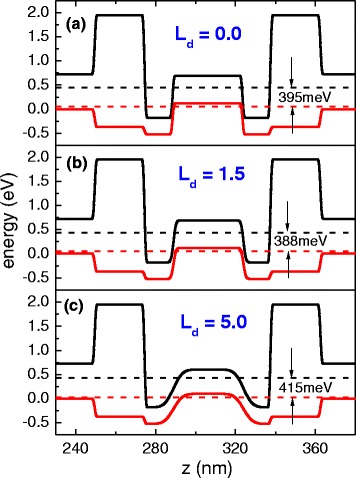
QW profiles for the nominal parameters of sample A (**a**), slightly modified profile of sample A (**b**), and strongly modified profile proposed for sample B (**c**)

Considering EDX profiles showing interface changes for sample B, we propose the following changes of the QW potential shape for the investigated samples. The composition profile of the sample with interface intermixing at the boundaries of the quaternary layer has been modeled by the Gauss error function [28, 29]—typically used to describe interdiffused interfaces. Figure 5 shows changes of the QW shape for three different values of the effective diffusion lengths *L*_d_ (which is not exactly known for our structures). *L*_d_ is tuned in a way to get the best agreement of the energy level spacing and outgoing optical transitions with those observed in the experiment as seen in Fig. 2. The concentration profile of groups III and V in the QW is given by:

$$ w(z)={w}_2-\frac{w_2-{w}_1}{2}\left[\mathrm{e}\mathrm{r}\mathrm{f}\left(\frac{L+2z}{4{L}_{\mathrm{d}}}\right)+\mathrm{e}\mathrm{r}\mathrm{f}\left(\frac{L-2z}{4{L}_{\mathrm{d}}}\right)\right] $$

where *w*_1_ and *w*_2_ are the initial concentrations of atoms in the well and in the barrier, respectively, *L* is the as-grown nominal width of the QW, *z* is the quantization direction along the growth axis (QW centered at *z* = 0), and “erf” denotes the error function. For sample A, small modifications of the interfaces (e.g., *L*_d_ = 1.5 nm) do not change the optical transition energies significantly and the agreement with the experiment remains good—see Table 1 (i.e., the correction due to the inclusion of intermixing is of the order of a few millielectronvolts). For sample B, a much stronger modification had to be used (*L*_d_ = 5 nm) to obtain agreement with the experimental value (see Table 1).

**Table 1:** Experimental and calculated fundamental type II transition energies for two samples with As content in W-design GaSb/AlSb/InAs/Ga_0.665_In_0.335_As_x_Sb_1 − x_/InAs/AlSb/GaSb QWs equal to 0 and 10 %, respectively

Sample	As content (%)	Experiment (eV)	Calculated—nominal (eV)	Calculated—intermixing (eV)
A	0	0.386	0.395	0.388
B	10	0.417	0.388	0.415

This confirms that the stronger intermixing in the As-containing structures can indeed cause a blueshift of the optical transition instead of the redshift which would be expected for rectangular—or less interdiffused—structures. The intermixing might be due to the finite time (roughly 2 s) that the As valve takes to change its position, and hence the flux, after the first InAs layer and before the second InAs layer. This causes some gradient in As concentration, which can be understood as intermixing. Any attempts of using shutter sequencing combined with growth interruptions within the W-QW have led to samples that did not exhibit any PL signal. Summarizing, we have shown that interface quality has a significant impact on the energy structure in our type II quantum wells, similarly as to what was previously shown for other low dimensional systems like, e.g., type I quantum wells [28], superlattices [30], and quantum dots [31].

## Conclusions

In conclusion, type II “W”-shaped GaSb/AlSb/InAs/GaIn(As)Sb/InAs/AlSb/GaSb QWs have been investigated. Optical spectroscopy (photoluminescence and photoreflectance) combined with band structure calculations made in the eight-band k·p formalism has been used to investigate the fundamental optical transition. These have been supported by HAADF-STEM images and EDX analysis in association with the numerical modeling of interface intermixing to show the changes in QW element compositions and prove an apparent arsenic effect on the structural and optical properties of type II “W”-shaped QW with quaternary GaInAsSb layers. We have shown that incorporation of arsenic into the GaInSb hole-confining layer does not only change the composition-driven energy gap and strain but also has a strong impact on the InAs/GaInAsSb interface. This affects the electronic structure of the quantum well to such an extent that it leads to an increasing blueshift of ground state type II transition with the increase of arsenic content in this layer. These results indicate that this effect should be taken into account when considering such QWs for the design of active regions of improved performance interband cascade lasers and that additional work on the technological side is necessary to minimize interface diffusion when GaInAsSb layers are utilized.

## Abbreviations

EDX: Energy-dispersive X-ray spectroscopy
HR-XRD: High-resolution X-ray diffraction
PL: Photoluminescence
PR: Photoreflectance
QWs: Quantum wells
STEM: Scanning transmission electron microscopy
